# Stereotactic Radiotherapy for Brain Metastases: Imaging Tools and Dosimetric Predictive Factors for Radionecrosis

**DOI:** 10.3390/jpm10030059

**Published:** 2020-07-04

**Authors:** Marco Lupattelli, Emanuele Alì, Gianluca Ingrosso, Simonetta Saldi, Christian Fulcheri, Simona Borghesi, Roberto Tarducci, Cynthia Aristei

**Affiliations:** 1 Radiation Oncology Section, Perugia General Hospital, 06022 Perugia, Italy; mlupattelli62@gmail.com (M.L.); saldisimonetta@gmail.com (S.S.); 2 Radiation Oncology Section, Department of Surgical and Biomedical Science, University of Perugia, 6100 Perugia, Italy; emanuele.ali.06@gmail.com (E.A.); cynthia.aristei@unipg.it (C.A.); 3 Medical Physics Department, Perugia General Hospital, 06022 Perugia, Italy; cplfulcheri@gmail.com (C.F.); roberto.tarducci@gmail.com (R.T.); 4 Unit of Radiation Oncology, S.Donato Hospital, 20097 Arezzo, Italy; s.borghesi@gmail.com

**Keywords:** radionecrosis, stereotactic radiotherapy, imaging tools, dosimetric parameters

## Abstract

Radionecrosis (RN) is the most important side effect after stereotactic radiotherapy (SRT) for brain metastases, with a reported incidence ranging from 3% to 24%. To date, there are no unanimously accepted criteria for iconographic diagnosis of RN, as well as no definitive dose-constraints correlated with the onset of this late effect. We reviewed the current literature and gave an overview report on imaging options for the diagnosis of RN and on dosimetric parameters correlated with the onset of RN. We performed a PubMed literature search according to the preferred reporting items and meta-analysis (PRISMA) guidelines, and identified articles published within the last ten years, up to 31 December 2019. When analyzing data on diagnostic tools, perfusion magnetic resonance imaging (MRI) seems to be very useful allowing evaluation of the blood flow in the lesion using the relative cerebral blood volume (rCBV) and blood vessel integrity using relative peak weight (rPH). It is necessary to combine morphological with functional imaging in order to match information about lesion morphology, metabolism and blood-flow. Eventually, serial imaging follow-up is needed. Regarding dosimetric parameters, in radiosurgery (SRS) V12 < 8 cm^3^ and V10 < 10.5 cm^3^ of normal brain are the most reliable prognostic factors, whereas in hypo-fractionated stereotactic radiotherapy (HSRT) V18 and V21 are considered the main predictive independent risk factors of RN.

## 1. Introduction

Brain metastases represent the most frequent intracranial tumor with an incidence of up to 35%. They considerably affect quality of life in cancer patients because of the onset of several symptoms, such as seizures, focal neurological deficits or signs of intracranial hypertension, based on location and size [1]. Radiotherapy has an important role in the management of brain metastases. Stereotactic radiotherapy (SRT) is a well-established treatment for patients with brain metastases, which may be delivered in a single fraction (radiosurgery, SRS) or in a few fractions (hypo-fractionated stereotactic radiotherapy, HSRT) [2,3]. SRT is an ablative treatment based on the delivery of high doses per fraction to the target, and one of the most important and serious side effects in the brain is represented by radionecrosis (RN), with a reported incidence in the literature ranging from 3% to 24% [3,4,5,6]. RN onset usually occurs after 3–12 months from radiation therapy, but it can also occur in subsequent years. From the physio-pathological point of view, RN seems to be due to a tissue reaction to radiotherapy with disruption of the blood-brain barrier, which leads to an increased capillary permeability and extra-cellular edema. In addition, areas of necrosis and fibrinous exudate appear, with vessel thickening, thrombosis and intravascular occlusion [3,6]. RN can be asymptomatic or it can cause several non-specific symptoms such as seizures, cognitive deficits and nausea, depending on the specific irradiated area. From the clinical point of view, RN is a multifactorial event depending on radiotherapy dose, prescription isodose, treatment technique, volume of target, site of irradiation, and volume of brain irradiated [7]. It is difficult to delineate the risk of RN given the variability in patient and disease factors. However, several authors have clearly demonstrated that in stereotactic radiotherapy RN is correlated with dosimetric parameters such as total delivered dose, and with volume of normal brain receiving a specific set of Vx (percentage of organ at risk (OAR) volume receiving at least the x dose) [7,8,9,10]. An important issue related to RN is the differential diagnosis with tumor progression, which is a key challenge for the management of patients who have undergone SRT for brain metastases. In fact, RN can mimic a disease progression showing an increase of the irradiated lesion with peripheral contrast enhancement and perilesional edema. Systemic therapy with corticosteroids (dexamethasone 2–8 mg, daily) is used to reduce perilesional edema. Anticoagulants, hyperbaric oxygen therapy, and bevacizumab can be considered, but the role of these therapies has yet to be defined.

Conventional imaging such as computed tomography (CT) and magnetic resonance imaging (MRI) are used in clinical practice to discriminate between RN and in-field recurrence after stereotactic radiotherapy. Advanced MRI tools and molecular imaging modalities (e.g., positron emission tomography) seem to be helpful in the definition of RN areas within the irradiated tissue. The aim of the present work is to highlight the available imaging tools in clinical practice for the diagnosis of radionecrosis and to analyze dosimetric parameters that might influence the onset of RN in patients treated with SRT for brain metastases.

## 2. Materials and Methods

We reviewed the current literature and gave an overview report on imaging options for the diagnosis of RN and on dosimetric parameters correlated with the onset of RN in patients affected by brain metastases treated with stereotactic radiotherapy. We performed a PubMed literature search according to the preferred reporting items and meta-analysis (PRISMA) guidelines [11]. We identified articles published within the last ten years, up to 31 December 2019, using Medline search with the following selection criteria: English language, full papers, diagnosis of brain metastases, and treatment of brain metastases with stereotactic radiotherapy. The following Medline terms were used: brain metastases, radionecrosis, pseudotumor, MRI, positron emission tomography (PET), metastasis-directed radiotherapy, stereotactic radiotherapy, radiosurgery, stereotactic ablative radiotherapy, hypo-fractionated stereotactic radiotherapy, fractionated stereotactic radiotherapy. Two authors (E.A and M.L.) independently performed the study selection. Disagreements were resolved by consensus with two other authors (G.I. and C.A.). We reviewed the full version of each article.

## 3. Results

### 3.1. Diagnostic Imaging Tools in Radionecrosis

Biopsy of the suspected lesion and its subsequent histological analysis is the best method to distinguish between RN and tumor progression and currently this represents the gold standard. Nevertheless, biopsy may be difficult not only because it is an invasive procedure but also because of the frequent residual tumor cell mix and necrosis in surgical specimens [12]. More specifically, RN usually causes hypocellularity in necrotic areas with foamy macrophages and hemosiderophages, while in tumor progression there is an increased cellularity with strong nuclear pleomorphism [13]. The coexistence of these two conditions makes differential diagnosis very challenging. The use of new imaging tools might allow non-invasive differential diagnosis. To date, there are still no unanimously accepted criteria for iconographic RN diagnosis, but its differentiation from tumor progression is extremely important because of different therapeutic approaches and prognosis [3,12,13]. Conventional imaging represented by MRI and CT are the most commonly used methods for the follow up of patients with brain metastases. Both RN and tumor recurrence can appear in MRI as an enlargement of an enhancing centrally necrotic lesion in T2-weighted sequence with associated surrounding edema and generally mass effect. A useful information to distinguish between these two pathological entities can be the time elapsed since radiotherapy because RN occurs at least three months after irradiation. Other features that suggest RN presence are the involvement of periventricular white matter, the appearance of an enhancing area with a “Swiss cheese” or “soap bubble” aspect and the volume increase of the lesion with a subsequent spontaneous shrinkage without any anticancer treatment [14]. To assess quantitatively the information on MRI, Daquesada et al. in a radiographic-pathological study evaluated a numeric parameter named “lesion quotient” (LQ) to discriminate between RN and tumor progression. This is defined as the area of a lesion hypo-intensity on a T2-weighted MR image divided by its area on a contrast-enhanced T1- weighted image. In RN, LQ has been proposed to be less than 0.3 and in tumor recurrence greater than 0.6; values ranging from 0.3 to 0.6 have been considered for a combination of RN and tumor persistence. The authors found a high predictive LQ value with high sensitivity and specificity for RN but not for pure tumor persistence or for a combination of the two [15]. This parameter requires further validation considering the conflicting results reported in the literature [16].

### 3.2. Diffusion and Perfusion MRI

**Figure 1:**
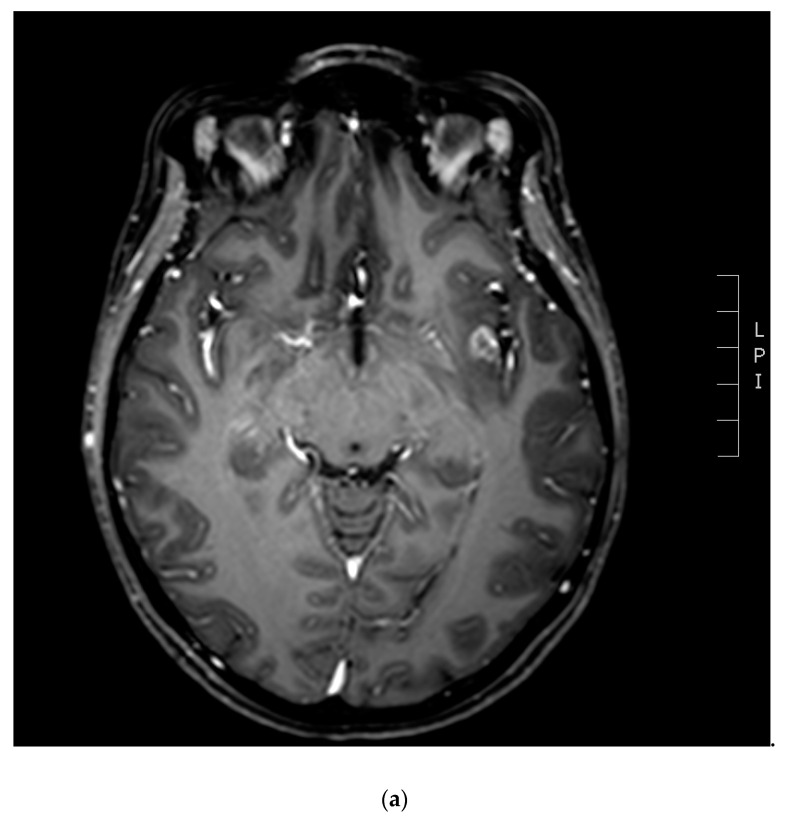

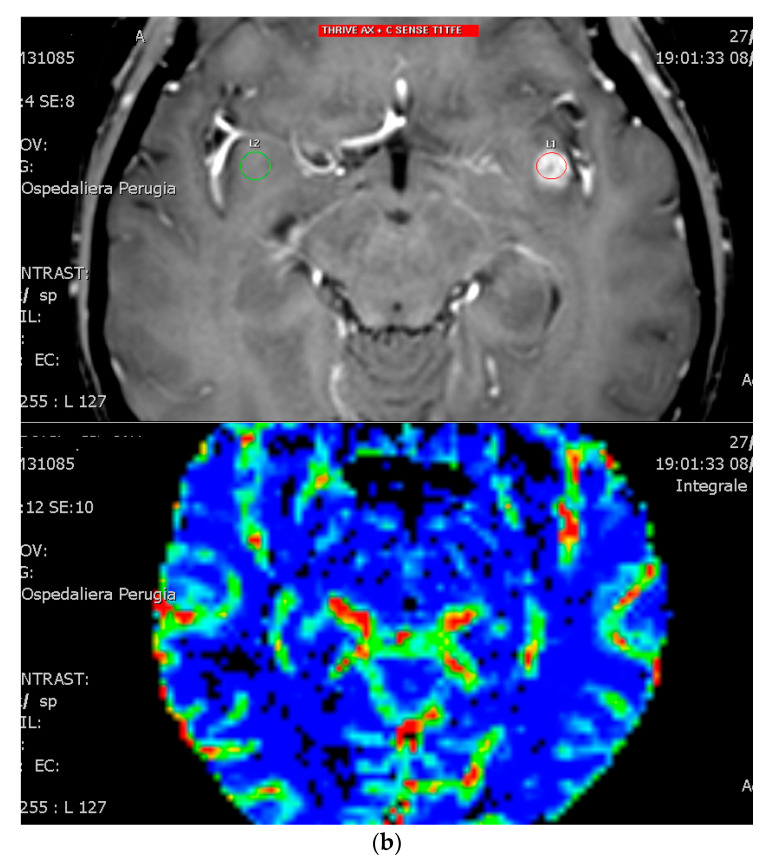
(**a**) Contrast enhanced T1-weighted magnetic resonance imaging (MRI) showing a suspected in-field recurrence one year after SRS (20 Gy) for a breast cancer metastasis in the left insular cortex; (**b**) the perfusion study is suggestive of disease progression because of relative cerebral blood volume (rCBV) increase due to the neo-angiogenesis within the insular cortex tissue.

MRI has several limitations related to the frequent overlapping between tumor and necrosis radiological characteristics [15]. For this reason, functional imaging has aroused great interest and it has been studied widely for differential diagnosis. In particular, diffusion and perfusion MRI have been evaluated. The most frequently analyzed parameter in diffusion is apparent diffusion coefficient (ADC), a measure of the water molecules diffusivity within each voxel, which depends on cellularity and on the presence of intracellular structures that slow down the water molecules’ movement. In tumor tissue, characterized by a high cellularity, diffusion restriction leads to a diffusion signal increase and in turn to a signal intensity decrease in ADC images. In radionecrotic areas, there is a diffusion signal decrease due to the movement of water molecules leading to a signal intensity increase in ADC images. However, in clinical practice this distinction is not so clear and immediate due to confounding factors such as infiltration and proliferation. Nevertheless, ADC values can provide useful information to make a differential diagnosis [12,14]. Another parameter that can be evaluated in diffusion imaging is fractional anisotropy (FA) which describes the directional selectivity of the random water molecules’ diffusion in tissue [17]. Theoretically, FA value should be lower in RN than in the tumor because of the disruption of normal axonal organization and of supporting cells in the latter. To date, the role of FA is investigational with conflicting results in the literature [18,19,20]. The role of perfusion MR has also been investigated because it provides information about tumor vascularity (Figure 1). Relative cerebral blood volume (rCBV) is the most frequently used parameter in perfusion and it has a higher value in tumor recurrence than in RN, because of the neo-angiogenesis within the tumor tissue [12,14,21]. Mitsuya et al. in their analysis proposed a value of 2.1 as rCBV threshold with high sensitivity and specificity, although they suggested that these values have to be used with caution [22]. The maximal change in signal intensity during contrast enhancement measured by the relative peak weight (rPH) is strictly related to rCBV, showing higher values in tumor recurrence than in RN. A third parameter frequently used in perfusion imaging is the percentage of signal-intensity recovery (PSR), which reflects the blood-brain barrier integrity when measuring the contrast leakage. Tumor recurrence is characterized by recruitment of abnormal and leaking vessels; hence, PSR is lower in tumor recurrence compared with RN. Barajas et al. reported values >76.3% as a PSR cut-off to define RN, with high sensitivity and specificity [23]. Information on the metabolic compositions within the tissue by magnetic resonance spectroscopy (MRS) could be useful in making differential diagnosis. The main metabolites analyzed in the literature are *N*-acetyl-aspartate (NAA), choline (Cho), creatine (Cr), lipid and lactate, and resulted that the Cho-Cr ratio and Cho-NAA ratio are higher in tumor recurrence than in RN. An elevated lipid-lactate peak seems to be more typical of RN although there may be necrosis and tumor coexistence that acts as a confounding factor. To date, no study in the literature strictly supports MRS utility and reliability [12,14,21,24].

### 3.3. Nuclear Medicine Imaging

Regarding nuclear medicine imaging, PET has been widely evaluated. The most commonly studied tracer is fluorodeoxyglucose (FDG) but its use in differentiating tumor recurrence from RN is limited by FDG uptake in the normal cortex, which reduces the accuracy of the distinction between the two pathological entities. Many efforts have been made to evaluate other radiotracers such as 3,4-dihydroxy-6-(18)F-fluoro-l-phenylalanine ((18)F-FDOPA), *O*-(2-[18F]fluoroethyl)-L-tyrosine (18F-FET), and carbon-11-labeled methionine (11C-MET). In particular 11C-MET seems to be a very interesting tracer. As protein synthesis is strictly related to cellular proliferation, tumor 11C-MET concentration is generally higher than in the healthy brain. Its uptake occurs through active transport into tumor cells independently from sodium channels and it depends on the concentration gradient reflecting intracellular amino acid metabolism. In RN, there is a passive diffusion of 11C-MET in the extracellular space due to the blood-brain barrier disruption. Hence, in tumor recurrence 11C-MET uptake is usually higher than RN. Glaudemans et al. stated that 11C-MET-PET is useful in the identification of tumor recurrence after radiation, showing a diagnostic accuracy of between 82% and 94% [25]. In order to differentiate between tumor recurrence and RN, Garcia et al. quantified 11C-MET-PET data (SUV max/SUV mean background) in patients affected by high-grade glioma and treated with post-operative radiotherapy. They found that SUV lesion/background was 2.79 ± 1.35 in tumor recurrence, and 1.53 ± 0.39 in radionecrosis (*p* < 05) [26]. However, MET-PET might also give false positive and false negative. In conclusion, biopsy represents the gold standard to make differential diagnosis. However, it is not always feasible and multimodal imaging is often required. Morphological imaging is inadequate to differentiate between tumor recurrence and RN after radiotherapy. It is necessary to combine morphological with functional imaging in order to match information about lesion morphology, metabolism and blood-flow. Eventually, serial imaging follow-up is needed [12,27].

### 3.4. SRS Dosimetric Parameters Related with RN

Stereotactic radiosurgery is a feasible and effective treatment for brain metastases with excellent local control rates (70–90%) [28], but it is not suitable for all patients. For instance, those treated with SRS for larger lesions (>2 cm) are at high risk of developing neurological complications [29]. Brain necrosis is the most important late toxicity reported, leading to neurological complications in 2–32% of patients [30]. RN may be symptomatic or asymptomatic, and in the latter case the diagnosis comes from follow-up brain MRI. The rate of RN (symptomatic and asymptomatic) has been reported in up to 50% of SRS irradiated brain metastases, and its onset has been correlated with SRS dose, tumor volume and location of the lesion [31,32,33]. Flickinger et al. [7] demonstrated that the risk of RN is influenced by the treated volume and by the total dose delivered. In the RTOG dose-escalation protocol 90–05, SRS doses were investigated in patients previously treated with whole brain radiation therapy (WBRT), reporting values of 24 Gy, 18 Gy and 15 Gy for lesions ≤ 2 cm, 2.1–3 cm and 3.1–4 cm, respectively [34]. Several authors tried to identify predictive factors of RN in order to reduce its occurrence. Dosimetric parameters have been widely investigated (Table 1), and many studies have correlated the risk of RN with normal brain volume receiving a specific dose of 10 and 12 Gy (V10 and V12). Korytko et al. [32] reported a 20% risk of symptomatic RN for V12 values between 5 and 10 cm^3^ but this correlation was not significant for asymptomatic RN. In a subsequent series of about 173 lesions treated with a median dose of 18 Gy, the multivariate analysis identified V10 and V12 as the most significant predictive factors of RN with a risk of 34% for V10 > 10.4 cm^3^ and for V12 > 7.8 cm^3^ [33]. In 2011 Minniti et al., evaluating 310 brain metastases treated with LINAC-based SRS, reported a RN rate of 24%. The multivariate analysis evidenced the close correlation between normal brain volume receiving a dose of 10 and 12 Gy and the development of RN, with a risk > 10% for V12 > 8.5 cm^3^ and a risk of 24% for V10 > 10.2 cm^3^ [28]. Sneed et al. analyzed 435 patients treated with gamma-knife for 2200 brain metastases. With a median lesion-imaging follow-up of 9.9 months, the one-year probability of neurological complications was 14% for metastases with a diameter from 2.1 to 5.1 cm, and the one-year probability of symptomatic RN was about 13% for V12 > 3.3 cm^3^ and V10 > 4.3 cm^3^. The median time of RN onset was 7.2 months [35].

Few studies have analyzed the possible correlation between the location of the metastasis and the risk of RN. In a retrospective series of 131 brain metastases treated with SRS, three location grades (LG) were defined based on the lesion depth from the brain surface. Grade 1 (superficial) included metastases located within 5 mm from the cortex, grade 2 (deep) for those at more than 5 mm from the cortex, and grade 3 (central) for lesions in the brainstem, cerebellar peduncle, diencephalon or basal ganglion. The multivariate analysis showed that LG (central location) and V22 > 2.6 cm^3^ were predictive factors for RN [36]. Regarding the volume of the target as a predictive factor for RN, Koutek et al. [37] in their retrospective study of 271 metastases treated with LINAC-based SRS demonstrated that the maximum tumor diameter on pre-SRS MRI was strongly correlated with RN. More specifically, they reported one-year RN rates of 2.9% in tumors with a diameter ≤ 0.5 cm, 6.6% in tumors between 0.6 and 1.0 cm, 19.1% in tumors between 1.1–1.5 cm and 37.8% in case of tumors ≥1.5 cm.

### 3.5. HSRT Dosimetric Parameters Related with RN

Hypo-fractionated stereotactic radiotherapy is based on the delivery of high total doses in few (2–5) fractions, combining the advantage of giving a large dose to the target volume with the radiobiological features of fractionation [38]. The radiobiological rationale of HSRT relies on the evidence that large tumors contain a proportion of radioresistant hypoxic cells [39]. The process of redistribution and reoxygenation within the tumor, taking place between dose fractions, enhances cell killing in the hypoxic area. Moreover, the linear-quadratic model of cellular survival evidences that late responding healthy tissues are better spared by a fractionated regimen than by a single acute dose, for a given level of tumor damage. In clinical practice, HSRT is a treatment modality for metastatic lesions in critical regions such as brainstem and for large volume targets (>10 cc), and it is characterized by a low toxicity profile and high rates of tumor local control [40]. Regarding toxicity after HSRT, several authors have demonstrated that delivering total doses of 24–35 Gy in 3–7 fractions to patients affected by oligo brain metastases exposed them to a RN risk of about 2–10% [41,42,43,44,45,46]. As the most important side effect of brain metastases HSRT represented by RN, many efforts have been made to identify useful dose constraints that might be predictive of normal brain damage (Table 2). Ernst-Stecken et al. [42] analyzed the risk of RN correlated with brain metastases HSRT to total doses of 30–35 Gy in five fractions. They found that a volume of normal brain >23 cm^3^ receiving more than 4 Gy per fraction is correlated with the onset of RN (risk of 70% for V20 > 23 cm^3^ compared with 14% for V20 < 23 cm^3^, *p* = 0.001). Fahrig et al. [43] evaluated three different HSRT fractionation schemes, in 150 patients with 228 brain metastases: 10 × 4 Gy, 7 × 5 Gy, and 5 × 6–7 Gy. Treatment-related toxicity was influenced by dose fractionation and volume of the lesion. For metastases > 15 cm^3^ (diameter > 3 cm) 10 × 4 Gy was better for acute and late complications but with lower tumor control rate compared with the other fractionation schemes. In a retrospective series of 78 patients with brain metastases in critical regions who underwent cyber-knife-based HSRT (31 Gy in five fractions), Inoue et al. [47] found that V14 > 7 cm^3^ was strongly correlated with the risk of RN. Minniti et al. [46] reported V24 > 16.8 cm^3^ and V21 > 20.9 cm^3^ as dose constraints correlated with RN in patients irradiated to a total dose of 27 Gy in 3 fractions. In a series on 289 patients treated with HSRT (3 × 9 Gy) the authors demonstrated that for V18 > 30.2 cm^3^ the RN rate was 14% [48]. More specifically, the estimated risk of RN was 0% for V18 < 22.8 cm^3^, 6% for 22.8 cm^3^ < V18 < 30.2 cm^3^ and 24% for V18 > 41.2 cm^3^. Eventually, comparative analysis between patients showed that those treated with SRS had a statistically significant higher rate of RN compared with HSRT patients (20% vs. 8%, *p* = 0.004). Comparing the risk of RN between SRS (20 Gy) and HSRT (6 × 6 Gy) in 98 patients affected by brain metastases, Kim et al. [44] reported a rate of 5% in patients treated with HSRT and of 17% in those treated with SRS (*p* < 0.05). In a series of 260 patients affected by 1–3 brain metastases, SRS (20 Gy) was associated with a higher risk of late toxicity (14%) compared with SRT (2% for a schedule of 25 Gy in five fractions) [45]. A recent systematic review has dealt with the problem of fractionation considering schedules of 2–5 fractions compared with the single fraction, reporting a one-year local control rate of 87.3% vs. 80% (I^2^ = 70.72%), whereas data on RN were inconclusive [49]. The results of this systematic review reflect the huge heterogeneity of data in the literature on fractionation schedules, volumetric data and dosimetric parameters analyzed. It seems that the dose received by the normal brain is not always a risk factor for RN. In a recent report on post-operative HSRT (25–35 Gy in 5 fractions) in 55 resected brain metastases, no association was found between RN and volumetric data evaluated, in particular V25, V30 and V35 [50]. The authors demonstrated that 105%, 110%, and 111% hotspots within the PTV are associated with RN, with HRs of 3.64, 8.47, and 6.90, respectively (*p* = 0.029, *p* = 0.04, and *p* = 0.038).

## 4. Conclusions

Many studies have focused on RN after stereotactic radiotherapy for brain metastases, trying to identify reliable diagnostic tools and specific predictive dosimetric factors. Regarding diagnosis, the biopsy of the suspected lesion is the gold standard but this is not always feasible and its interpretation may be sometimes difficult. Hence, serial morphological and functional follow-up imaging is of paramount importance. To date, perfusion-MRI seems to be very useful allowing the evaluation of blood flow in the lesion using the rCBV and of blood vessel integrity using the rPH. These parameters in combination with morphological MRI help to make differential diagnosis between RN and tumor progression [21,22]. In the near future, radiomics (based on computer-extracted texture features) identifying MRI quantitative patterns will provide diagnostic information not visually appreciable that will help to define the imaging phenotype of the analyzed lesion. For instance, Hettal et al. [52] assessed the clinical relevance of 1766 MRI radiomics features for differential diagnosis between RN and tumor progression, in patients treated with stereotactic radiotherapy for brain metastases. They found a prediction accuracy of 75% for radionecrosis, and of 91% for disease progression. Validation studies on radiomics and machine learning are needed to translate in-silico results into clinical practice.

RN onset is related to many factors but the most important seem to be the volume of the irradiated lesion, total dose and fractionation and the volume of brain receiving the specific dose. More specifically, the latter parameter has been widely investigated. Regarding HSRT, there is no consensus about dosimetric parameters mainly because of the heterogeneity of treatment schedules. Furthermore, few data are available in literature about HSRT compared with SRS. In HSRT, V18 and V21 are considered the main predictive independent risk factors of RN in three-fractionated schedules. In order to reduce RN onset, we suggest the following dose-constraints for normal brain: V18 < 30.2 cm^3^ and V21 < 20.9 cm^3^ [48]. Regarding SRS, V12 < 8 cm^3^ and V10 < 10.5 cm^3^ of normal brain seems to be the most studied and reliable dose-constraints [28,33]. Regarding the volume of the irradiated lesions, HSRT should be preferred for lesions with a diameter > 2 cm as well as for metastases located in central position (brainstem, cerebellar peduncle, diencephalon or basal ganglion).

## Figures and Tables

**Table 1:** Stereotactic radiosurgery.

	No of pts/no of Lesions	Location	Median PTV (Range) [cm^3^]	Median Total Dose (Range) [Gy]	V12 Value/Risk of RN	V10 Value/Risk of RN	Isodose Line Prescription	Location Grade/Risk of RN	Median Follow-Up (mo)	RN	S-RN
Minniti 2011 [28]	206/310	NA	2.81 (0.2–23.7)	18 (15–20)	>8.5 cc/>10%	>10.2 cc/24%	80–90%	NA	9.4 (2–42)	24%	10%
Ohtakara 2012 [36]	57/131	NA	0.52 (0.03–8.73)	22 (11–27)	>8.4 cc (ROC analysis)	NA	80%	*p* < 0.001	18.2 (7–45.9)	15.3%	6.9%
Blonigen 2010 [33]	63/173	NA	Mean 0.52	18 (12–22)	>7.8 cc/34%	>10.4 cc/34%	80%	NA	13.7 (3.5–51)	14%	10%
Kohutek 2015 [37]	160/271		0.5 (0.04–47)	21 (15–22)	NA	NA	80%	NA	17.2 (1.7–67.9)	25.8%	17.3%
Korytko 2006 [32]	129/198	NA		17.3 (11–25)	5–10 cc/20%	NA	50%	NA	NA	NA	NA

PTV: planning target volume; RN: radionecrosis; s-RN: symptomatic radionecrosis; NA: not available.

**Table 2:** Hypo-fractionated stereotactic radiotherapy.

	No of pts/no of Lesions	Median PTV (Range) [cc]	Median Total Dose (Range) [Gy]	V18 Value/Risk of RN	V21 Value/Risk of RN	V4 Value/Risk of RN	Isodose Line Prescription	Median Follow-Up (mo)	RN	S-RN
Minniti 2016 [48]	138/289	17.9 (5.6–54)	27 (9 × 3)	≤30.2/5%; >30.2/14%	NA	NA	80–90%	29	2.89%	5%
Minniti 2014 [46]	135/171	16.4 (3.4–62.7)	27 (9 × 3) (≥2 cm); 36 (12 × 3) (<2 cm)	≥26.2/14%; <26.2/4%	≥20.9/14%; <20.9/4%	NA	80–90%	11.4	9% (1 yr); 18% (2 yr)	NA
Doré 2016 [51]	95/97	12.9 (0.8–64.7)	23.1 (7.7 × 3)	NA	Median 5 cc; *p* = 0.010	NA	70%	17 (0.6–76)	20.6%	NA
Ernst-Stecken 2006 [42]	51/2	13 (1.70–95.97)	30 (6 × 5); 35 (7 × 5)	NA	NA	>23 cc; *p* = 0.001	90%	7	NA	NA

PTV: planning target volume; RN: radionecrosis, S-RN: symptomatic radionecrosis.
